# Imprinting of cerebral cytochrome P450s in offsprings prenatally exposed to cypermethrin augments toxicity on rechallenge

**DOI:** 10.1038/srep37426

**Published:** 2016-11-17

**Authors:** Anshuman Singh, Anita Agrahari, RadhaDutt Singh, Sanjay Yadav, Vikas Srivastava, Devendra Parmar

**Affiliations:** 1 Developmental Toxicology Division, CSIR-Indian Institute of Toxicology Research, Vishvigyan Bhawan, 31 M.G. Marg, Post Box No. 80, Lucknow-226 001, Uttar Pradesh, India

## Abstract

Epigenetic studies were carried in the rat offsprings, born to dams treated with cypermethrin (orally; 5.0 mg/kg) from gestation day (GD) 5 to 21 and rechallenged with cypermethrin (orally; 10 mg/kg for 6 days), at adulthood (12 weeks) to understand the mechanism underlying the overexpression of cerebral cytochrome P450s (CYPs) in exposed offsprings. The data revealed alterations in histone H3 acetylation and DNA methylation in promoter regions of CYP1A- and 2B- isoenzymes in the brain isolated from rechallenged animals. Further, bisulphite sequencing revealed critical CpG methylation changes in BARBIE BOX (Barbiturate response element) and BTE (Basal transcription element) in promoter of CYP2B1 in the brain isolated from rechallenged animals. Western blotting and DNA laddering/fragmentation studies revealed a greater magnitude of increase in the signalling pathways associated with apoptosis in the rechallenged animals. The data have indicated that overexpression of cerebral CYPs could be due to the imprinting of CYPs. Further, increased apoptosis observed in the rechallenged offsprings has suggested that these epigenetic changes in CYPs may predispose the prenatally exposed offsprings to the neurotoxic effects of other centrally acting drugs and chemicals when subsequently rechallenged later at life.

Cypermethrin, a type II synthetic pyrethroid, has been widely used in home formulations and agricultural practices^1^. Cypermethrin is reported to have high affinity for the central nervous system (CNS) and the voltage gated ion channels are the primary target in both insects and mammals^2^. The toxicity of pyrethroids is determined to a large extent by its kinetics^3^. As the concentration of pyrethroids increases in the brain, so does the symptoms of neurobehavioral toxicity^4^. Studies from our laboratory have shown that neurobehavioral toxicity of type II pyrethroids is dependent on its metabolism, catalyzed by xenobiotic metabolizing cytochrome P450s (CYPs) in rat brain and liver^5^,^6^. Deltamethrin, another type II pyrethroid, was reported to induce the expression of CYP1A and 2B isoenzymes, involved in its toxicity in rat brain and liver^7^,^8^. The enrichment of these specific CYP isoenzymes in brain and liver was found to potentiate the neurobehavioral toxicity of pyrethroids^7^.

Pregnancy has been recognized as a potentially critical window of vulnerability of exposure to a variety of chemicals^9^. Recent studies from our laboratory has shown that prenatal exposure to low doses of cypermethrin and deltamethrin formulation induces over-expression of CYPs and neurotransmitter receptors and rate limiting enzymes involved in catalyzing specific neurotransmitters during development^10^,^11^,^12^,^13^. These alterations in CYPs and specific genes involved in neurotransmission processes were found to be associated with the neurobehavioral changes and accumulation of the parent compound and its metabolites in brain of the exposed offsprings^10^,^11^,^12^,^13^. Rechallenge of the offsprings at adulthood (12 weeks) was found to produce greater magnitude of alterations in the xenobiotic CYPs and genes involved in neurotransmission processes^12^,^13^. Previous study from our laboratory has also shown that rechallenge of young offsprings with lindane, an organochlorine, led to an increased incidence of convulsions in the prenatally exposed offsprings therefore suggesting that prenatal exposure of pesticides may lead to increased responsiveness of the CYPs in brain and liver in the exposed offsprings^14^. Computational sequence analysis from our laboratory indicating complete absence of short interspersed repeat elements (SINE) in the upstream, coding and downstream sequences of xenobiotic metabolizing CYPs have suggested the potential of these CYPs to be imprinted in offsprings isolated from mothers exposed to cypermethrin or other similar pesticides during gestation^11^. As pyrethroids, like other pesticides also possess endocrine disrupting properties^15^, hormonal imprinting may also play a role in the over-expression of CYPs in the prenatally exposed offsprings.

It is well established that histone acetylation as well as DNA methylation immensely contributes towards the maintenance of the imprinting status of the genes and these two epigenetic processes are dynamically linked^16^,^17^. Epigenetic modifications have been reported in the genes involved in memory formation and motor activity^18^. The involvement of xenobiotic metabolizing CYPs in memory formation and motor function prompted us to investigate the role of promoter methylation and histone acetylation in regulating the imprinting status of CYPs in prenatally exposed offspringsrechallenged at adulthood. To investigate if modifications in these epigenetic processes can predispose challenged offsprings towards apoptotic pathways, attempts were also made to study the apoptosis in rechallenged offsprings.

## Materials and Methods

### Animals and treatment

Adult male (~12 weeks old) and female (~10 weeks old) albino wistar rats of proven fertility were obtained from the Animal House facility of Indian Institute of Toxicology Research (IITR), Lucknow. All the animals were maintained on a commercial pellet diet and water *ad libitum* in a temperature-controlled room with a 12/12-h light/dark cycle and cared for in accordance to the policy laid down by Animal Care Committee of IITR, Lucknow. The animal experimentation was approved by the Ethical Committee of the Institute. The experimentations were carried out in accordance with the relevant guidelines of the Ethical Committee of the institute. Female rats were allowed to mate with adult males (3:1). On day 0 of pregnancy (confirmed by a positive vaginal smear), the rats were randomly divided into two groups. Animals in group 1 received 5.0 mg/kg body weight (bw) orallyofcypermethrin (technical grade, mixture of isomers, 98% pure) from gestation day (GD) 5 to GD21. Animals in group 2 served as control and received corn oil in an identical manner. On the day of parturition, the average litter size was adjusted to eight per dam in all the groups with equal number of males and females as far as possible.

The male offsprings born to the control or cypermethrin treated dams were divided into 4 groups of 15 offspring each at 12 weeks. The offsprings born to control dams received corn oil and served as control group. The offsprings born to cypermethrin treated dams also received corn oil and served as prenatally exposed group. The offsprings born to control dams received cypermethrin, 10 mg/kg bw, orally for six consecutive days and served as postnatally exposed group. The offsprings born to cypermethrin treated dams received cypermethrin, 10 mg/kg bw, orally for six consecutive days and served as rechallenged group. After sacrifice, brain was immediately removed and processed for isolation of microsomes, tissue lysate and DNA for western blotting and DNA laddering/fragmentation studies as described previously^10^,^11^,^12^,^13^,^14^,^19^. The antibodies used in the blotting studies were purchased from Abcam (USA) having ab196495(1:750), ab182733(1:500), ab62465(1:1000), ab2013(1:500), ab90363(1:500) and ab16039 (1:2000) as their product codes and ratios. These experiments were performed in triplicates from the brain samples isolated from three different rats.

The brain tissues were also processed for isolation of DNA by ZR Genomic DNA^TM^-Tissue Mini Prep kit (Zymo Research, USA) using the manufacturers protocol. This purified DNA was used to perform bisulphite conversion and PCR amplification for sequencing studies, for conduction of 5-methyl CpGChIP assay and histone-ChIP assay and also for methylation and un-methylation specific PCR. All experiments were performed in triplicates from the brain samples isolated from three different rats. The methodologies for these are described below.

### Bisulphite Conversion and sequencing

For bisulphite conversion, 800 nanogram of DNA was bisulphite converted using the EZ DNA Methylation Kit (Zymo Research, Orange, CA). PCR was carried out using primers (see Supplementary Table S1 for the primer sequences) designed by Methyl primer express software (Applied Bio systems). 1 μl (10–30 ng) of bisulphite converted DNA was PCR amplified under following cycling conditions: 95 °C for 3 min, 40 cycles of 95 °C for 30 s, 60 °C for 1 min, and 72 °C for 2 min, followed by one time delay cycle of 72 °C for 5 min and a 4 °C soak. Amplification of the target region was verified by gel electrophoresis on a 2% agarose gel. Samples were purified using the QiagenQiaquick gel extraction kit, quantified through Nanodrop spectrophotometer (ND-1000, Thermo Scientific) and sequenced using forward or reverse primers. Sequencing was done according to the previously reported method^20^. Results were analyzed using BIQ analyzer software^21^. These studies were performed in three replicates from the samples isolated from three different rat brains.

### ChIP Assay for analysis of Histone H3 acetylation

For ChIP based analysis for H3 acetylation, 25 mg of brain tissue was cross-linked in PBS containing 1.5% formaldehyde for 10 min at room temperature. Formalin was neutralized by adding 0.125 M glycine. This cross-linked tissue was homogenized in ChIPlysis buffer and incubated for 3 hours at 65 °C to lyse cells. Cell lysate was further sonicated to obtain fragment size between 300–1000 bases. After sonication, 10 μg of sonicated chromatin was incubated with 1 μg of pan-acetyl histone H3 antibody (Anti-Histone H3 (acetyl K9 + K14 + K18 + K23 + K27) antibody procured from Abcam, ab47915) or immunoglobulin G (IgG) control overnight at 4 °C. The DNA-antibody complex was then incubated for 2 hours with 40 μl of protein A/G agarose beads (Santa Cruz Biotechnology, USA, sc-2003) and precipitated by centrifugation. Precipitated DNA was repeatedly washed and digested with proteinase K and crosslinking was reversed at 65 °C for 4 hours. This was followed by phenol/chloroform extraction and ethanol precipitation of DNA. The DNA pellet was resuspended in TE (Tris-EDTA buffer) buffer and used for quantitative real time-PCR. Input DNA was also extracted similarly by phenol-chloroform method and used forreal-time polymerase chain reaction (qRT-PCR). The region selected for ChIPqRT-PCR was same as for MSP/UMSP (CYP1A1) or bisulphite conversion and sequencing (CYP2B1) (Tables S1, S2 and S3).

### ChIP assay for analysis of Methylated DNA

Genomic DNA from 25 mg of brain was extracted by ZR Genomic DNA^TM^- Tissue MiniPrep kit (Zymo Research) using the manufacturer’s protocol. 10 μg of this genomic DNA was diluted in Tris-EDTA (TE) and sonicated to obtain fragment size between 300–800 bases. After sonication, an aliquot was kept as input DNA. Remaining DNA was denatured for 10 min, rapidly cooled on ice and divided into 2 groups. 160 ng of sonicated DNA was incubated with protein A/G agarose beads + IgG and another with protein A/G agarose beads + 1 μg of anti-5-methyl cytosine antibody (monoclonal mouse, Zymo research) at 37 °C for 2 hrs in immunoprecipitation buffer. The DNA-antibody complex was then centrifuged and washed 3 times. The DNA precipitated by the antibody was digested with proteinase K, followed by phenol/chloroform extraction and ethanol precipitation. The DNA pellet was resuspended in TE buffer and used for qRT-PCR. The region selected for ChIPqRT-PCR was same as for MSP/UMSP (CYP1A1) or bisulphite conversion and sequencing (CYP2B1) (See Supplementary Tables S1, S2 and S2).

### Quantitative Real-Time ChIP-PCR (qRT-PCR)

The qRT-PCR was performed with DNA isolated from methyl chromatin immunoprecipitation (ChIP) and H3 Acetyl Histone ChIP. Input DNA and immunoprecipitated DNA were analyzed by qRT-PCR using primers specific for various CYPs reported in the study. After verification of melting temperature, the average threshold values (Ct) were used to calculate fold enrichment over isotype control. Triplicate reactions for gene of interest (CYP1A isoenzymes and CYP2B isoenzymes) were performed per sample. List of primers used is provided in the supplementary material (See Supplementary Table S2).

### Methylation specific PCR (MSP) and unmethylated specific PCR (UMSP)

The MSP and UMSP analysis was sperformed using bisulphite converted DNA and primers specific for either the methylated or unmethylated DNA. PCR conditions were same as for bisulphite PCR and sequencing. The region selected for MSP-UMSP was same as for ChIP assay for analysis of histone acetylation or DNA Methylation. The amplified products were run on a 2% agarose gel. Size was verified and quantified using Image J software from NCBI. List of primers used is provided in the supplementary material (See Supplementary Table S3).

### Statistical analysis

The data have been analyzed by one-way analysis of variance (ANOVA) followed by Newman–Keuls test for posthoccomparisons to all pair of columns between the groups using Graph Pad prism software (USA). Values up to *p < 0.05, **p < 0.01 and ***p < 0.001 have been considered to be significant. A power analysis was conducted using Post-hoc Power calculator at clincalc.com. The analysis revealed the statistical power of 0.8 for this study using alpha = 0.05 and beta = 0.2.

## Results

### Epigenetic Studies

#### Pan-acetyl H3 histone acetylation ChIP and qRT-PCR

**Figure 1:**
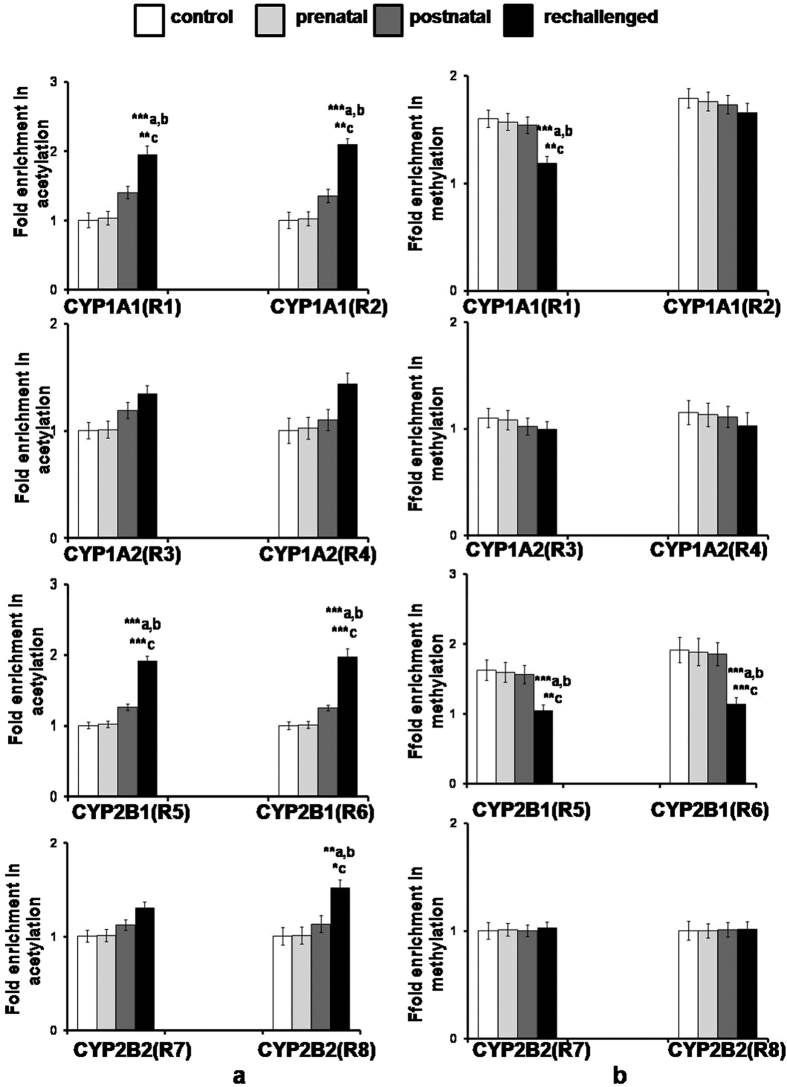
Histone H3 acetylation ChIP and Methylated DNA immunoprecipitation assay for analysis of acetylation and methylation in the promoters of CYP1A- and 2B- genes in cypermethrin exposed offsprings. (**a**) Histone H3 acetylation ChIP assay. a - compared to control group; b - compared to prenatally exposed offsprings group; c - compared to offsprings exposed postnatally with cypermethrin. (**b**) Methylated DNA immunoprecipitation assay. a - compared to control group; b - compared to prenatally exposed offsprings group; c - compared to offsprings exposed postnatally with cypermethrin. All the values represent mean ± S.E.M. of three experiments in each set (n = 3). Significant difference have been considered up to *p < 0.05, **p < 0.01 and ***p < 0.001.

Chromatin immunoprecipitation followed by qRT-PCR using primers specific for the promoter region of CYP1A1 or CYP2B1 showed no statistically significant changes in fold enrichment of histone acetylation in DNA extracted from brain isolated from control offsprings or control offsprings treated prenatally withcypermethrin or control offsprings treated postnatally at adulthood with cypermethrin (Fig. 1a). However, statistically significant change in fold enrichment of histone acetylation was observed using primers specific for CYP1A1 (R1 and R2) or CYP2B1 (R5 and R6) in brain isolated from prenatally exposed offsprings subsequently rechallenged with cypermethrin at adulthood. This increase in enrichment was found to be significant when compared to the control offsprings or prenatally exposed offsprings or control offsprings treated postnatally with cypermethrin (Fig. 1a). Similarly, primer pairs R3 and R4 of CYP1A2 or R7 and R8 of CYP2B2 showed no statistically significant change in fold enrichment of histone acetylation in DNA extracted from brain isolated from control offsprings or control offsprings exposed prenatally to cypermethrin or control offsprings treated postnatally at adulthood with cypermethrin. However, a statistically significant change in fold enrichment of histone acetylation was observed using primers specific for CYP2B2 (R8) in brain isolated from prenatally exposed offsprings subsequently rechallenged with cypermethrin. This enrichment was found to be significant when compared to the control offsprings or prenatally exposed offsprings or control offsprings treated postnatally with cypermethrin (Fig. 1a). No statistically significant changes in fold enrichment of histone acetylation was observed using primers specific for CYP1A2 (R3 and R4) or CYP2B2 (R7 and R8) in brain isolated from prenatally exposed offsprings subsequently rechallenged with cypermethrin when compared to the control offsprings or offsprings treated prenatally or control offsprings treated postnatally with cypermethrin (Fig. 1a).

#### Methylated DNA ChIP and qRT-PCR

ChIP assay with 5-methyl cytosine antibodies followed by qRT-PCR using primers specific for the promoter regions of CYP1A1 (R1 and R2). CYP1A2 (R3 and R4), CYP2B1 (R5 and R6) or CYP2B2 (R7 and R8) showed no statistically significant changes in fold enrichment of methylation in DNA extracted from brain isolated from control offsprings or control offsprings treated prenatally with cypermethrin or control offsprings treated postnatally with cypermethrin suggesting no CpG methylation changes in this region (Fig. 1b). However, significant decrease in DNA methylation was observed with primer pair R1 of CYP1A1 or primer pair R5 and R6 of CYP2B1 in the brain isolated from prenatally exposed offspringsrechallenged with cypermethrin. No decrease in DNA methylation was observed with primer pair R2 of CYP1A1 in brain isolated from rechallengedoffsprings suggesting no CpG methylation changes in this region (Fig. 1b). Likewise, no statistically significant changes in fold enrichment of DNA methylation was observed when primer pairs R3 and R4 of CYP1A2 or R7 and R8 of CYP2B2 were amplified with enriched DNA extracted from brain isolated from prenatally exposed offspringsrechallenged with cypermethrin at adulthood when compared to the control offsprings or offsprings treated prenatally with cypermethrin or control offsprings treated postnatally with cypermethrin (Fig. 1b).

#### MSP and UMSP Analysis of CYP1A1

**Figure 2:**
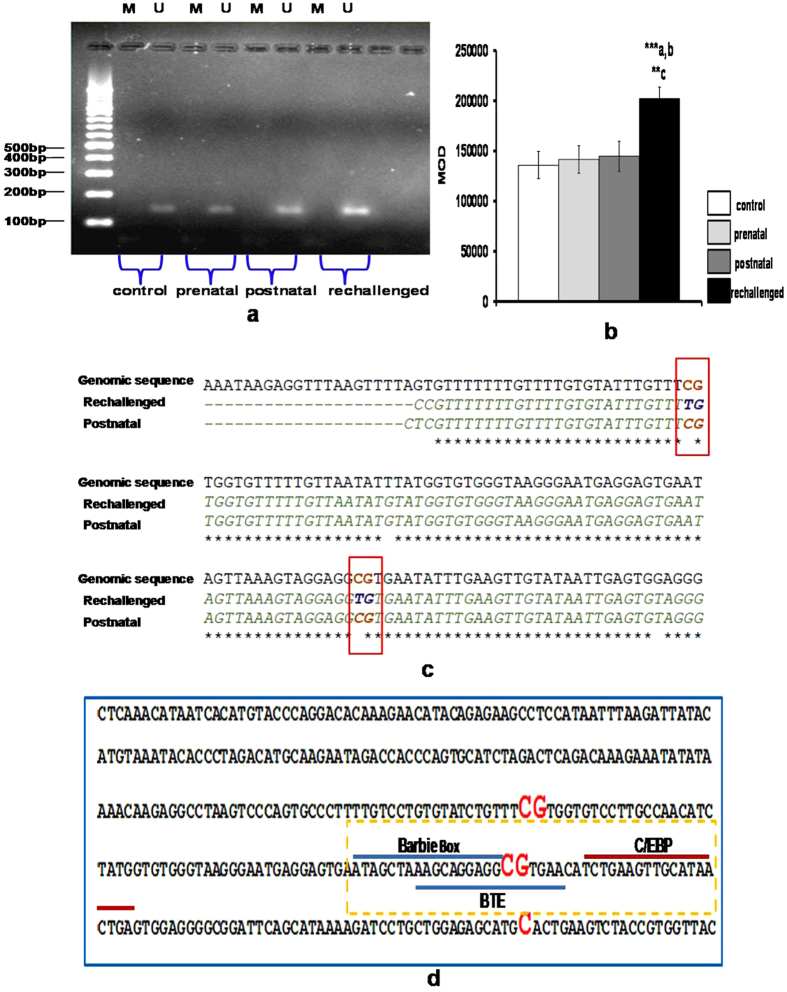
MSP, UMSP analysis and Bisulphite sequencing of CYP1A1 and CYP2B1. (**a**) Analysis of CYP1A1 promoter methylation through methylation specific-PCR inbrain of exposed offsprings. Bisulphite converted DNA was used for PCR with primers specific for both M (methylated CpG) and U (un-methylated CpG). (**b**) Densitometric analysis of UMSP (un-methylated DNA specific PCR) for CYP1A1 promoter in the brain samples. a - compared to control group; b - compared to prenatally exposed offsprings group; c - compared to offsprings exposed postnatally with cypermethrin. All the values represent mean × 10^−4^ ± S. E. M. × 10^−4^ of three experiments in each group. Significant difference have been considered up to *p < 0.05, **p < 0.01 and ***p < 0.001. (**c**) CYP2B1 promoter methylation in the brain of offspring which were either exposed only at adulthood (Postnatal) or exposed both during gestation period and at adulthood (Rechallenged) with cypermethrin. Bisulphite converted DNA was sequenced with promoter specific primers and analyzed using BIQ analyzer. The differentially methylated CGs is highlighted. (**d**) CYP2B1 proximal promoter sequence showing regulatory elements which could be affected by CG demethylation (dotted box). The CG elements showing demethylation in rechallenged group are highlighted. Single C in bold is the transcription start site of CYP2B1 promoter. Sites for barbiturate responsive element (BARBIE box), basal transcription element (BTE) binding and CCAAT-enhancer-binding proteins (C/EBP) are depicted.

As CYP1A1 showed significantly higher increase in expression, it was further analyzed by MSP and UMSP to validate the results obtained by ChIP assay. Using methylation specific primer sets for CYP1A1 (MSP), no amplification was observed in brain samples isolated from different groups. However in un-methylated specific PCR (UMSP), amplification was observed with one primer set of CYP1A1 (Fig. 2a and b). Densitometry analysis of bisulphite converted DNA using un-methylation specific primers (UMSP) for brain revealed significant increase in the amplified product in challenged offsprings when compared to prenatally exposed offsprings or control offsprings treated postnatally with cypermethrin or control offsprings (Fig. 2a and b).

#### Bisulphite sequencing for detecting methylation in CYP2B1

As specific MSP primers could not be designed for CYP2B1 due to the complexity of bisulphite converted DNA, bisulphite sequencing incorporating a wider range of promoter sequences was performed to understand the extent and dynamics of CpG methylation changes in CYP2B1. Primers covering approximately 300 bases upstream from the transcription start site (TSS) of CYP2B1 were chosen for amplification and sequencing from brain of different groups. Following sequencing, two CpGs at position −69 and −136 from the TSS were found to be hypomethylated in the brain of challenged offsprings (Fig. 2c and d). The hypomethylatedCpG at position −69 overlaps with barbiturate response element (BARBIE) box and BTE (basal transcription element) site of CYP2B1 promoter (Fig. 2c and d). No change in CpG methylation was found in other groups (data not shown).

### Apoptosis assessment in rechallengedoffsprings

#### DNA laddering/fragmentation analysis

**Figure 3:**
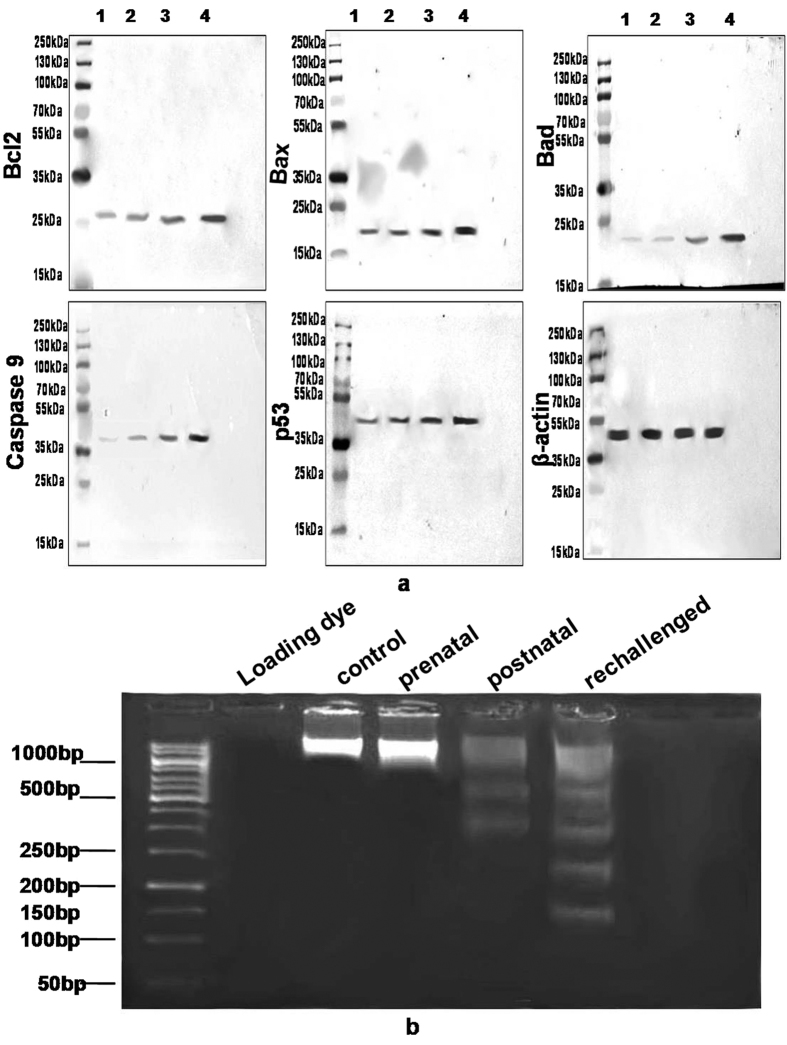
Western blots of anti- and pro-apoptoic proteins and DNA laddering/fragmentation. (**a**) Western blots of total protein isolated from brain of offsprings with anti-bcl2/bax/bad/caspase9/p53. Lane 1 contains protein from brain (50 μg) of rat offsprings raised on control mothers. Lane 2 contains protein prepared from brain (50 μg) of rat offsprings exposed prenatally to 5 mg/kg of cypermethrin. Lane 3 contains protein prepared from brain (50 μg) of rat offsprings exposed postnatally to 10 mg/kg of cypermethrin. Lane 4 contains protein prepared from brain (50 μg) of rat offsprings exposed prenatally to 5 mg/kg of cypermethrin and subsequently rechallenged with cypermethrin (10 mg/kg) at adulthood. (**b**) Representative image of DNA laddering/fragmentation.

DNA laddering/fragmentation analysis revealed no significant fragmentation in the DNA isolated from brain of prenatally exposed offspring when compared with control offsprings (Figs 3b and S1b). Less but significant DNA fragmentation was observed in the DNA isolated from brain of offsprings born to control dams treated with cypermethrin at adulthood when compared with control offsprings. Similarly, significant increase in the DNA fragmentation was observed in the DNA isolated from brain of prenatally exposed offspringsrechallenged with cypermethrin at adulthood when compared to the control offsprings.

#### Western blot analysis

Western blot analysis revealed no significant increase in the expression of Bcl-2, an anti-apoptotic protein, in brain lysate of prenatally exposed offsprings when compared with control offsprings. No significant increase was observed in the expression of Bcl-2 in brain lysate of offsprings born to control dams treated with cypermethrin at adulthood when compared with control offsprings. Similarly, no significant increase was observed in the expression of Bcl-2 in brain lysate of prenatally exposed offspringsrechallenged with cypermethrin at adulthood when compared to the control offsprings. Western blot analysis revealed no significant increase in pro-apoptotic Bad, caspase 9 and p53 in brain lysate of prenatally exposed offsprings when compared with control offsprings. No significant increase was observed in the expression of Bad, caspase 9 and p53 in brain lysate of offsprings born to control dams treated with cypermethrin at adulthood when compared with control offsprings. Significant increase was observed in the expression of Bad, caspase 9 and p53 in brain lysate of prenatally exposed offspringsrechallenged with cypermethrin at adulthood when compared to the control offsprings. Western blot analysis revealed no significant increase in the expression of Bax, and pro-apoptotic protein, in brain lysate of prenatally exposed offsprings when compared with control offsprings. A low but significant increase was observed in the expression of Bax in brain lysate of offsprings born to control dams treated with cypermethrin at adulthood when compared with control offsprings. A much higher and significant increase was observed in the expression of Bax in brain lysate of prenatally exposed offspringsrechallenged with cypermethrin at adulthood when compared to the control offsprings.

No significant increase in the Bax/Bcl-2 ratio was observed in brain lysate of prenatally exposed offsprings (0.87 ± 0.08; n = 3) when compared with control offsprings (0.72 ± 0.07; n = 3). Less but significant increase in the Bax/Bcl-2 ratio was observed in brain lysate of offsprings born to control dams treated with cypermethrin at adulthood (1.08 ± 0.07; n = 3) when compared with control offsprings (0.72 ± 0.07; n = 3). A much higher and significant increase in the Bax/Bcl-2 ratio was observed in brain lysate of prenatally exposed offspringsrechallenged with cypermethrin (1.55 ± 0.07) at adulthood when compared to the control offsprings.

## Discussion

Epigenetic studies have shown that greater magnitude of increase in the expression of cerebral CYPs in challenged offsprings is associated with the increase in HAT acetylation and decrease in DNA methylation. ChIP analysis showed significant increase in the fold enrichment of acetylated histone and significant decrease in the fold enrichment of methylated CpGs in the promoter regions of CYP1A1 and CYP2B1 in brain isolated from challenged offsprings when compared to control offsprings treated postnatally with cypermethrin or prenatally exposed offsprings. Suter *et al.*^22^,^23^ have earlier demonstrated that maternal tobacco exposure induces placental CYP1A1 expression which was associated with a decrease in promoter methylation at a critical xenobiotic response element (XRE) binding site^22^. It has been suggested that maternal smoking disturb placental methylation in a CpG site-specific manner which in-turn correlates with changes in the gene expression of phase I metabolizing enzymes such as CYP1A1^23^. Likewise, MSP-UMSP analysis indicating significant increase in the UMSP products of CYP1A1 in challenged offsprings has suggested an increased demethylation of specific CpGs in promoter regions of cerebral CYP1A1 in offspringsrechallenged with cypermethrin at adulthood.

Similarly, significant increase observed in histone acetylation in genomic DNA isolated from the brain of challenged offsprings may help in explaining the over-expression of CYP1A1 and CYP2B1 reported after prenatal exposure of cypermethrin and enhanced responsiveness of these CYPs in the challenged offsprings. It has been shown that a decrease in histone deacetylase I plays a central role in CYP1A1 expression, highlighting the requirement for an intensive sequence of chromatin remodelling events involved in the completion of the initial steps of Ah receptor mediated gene trans-activation^23^. Though not much information is available on the DNA methylation and histone acetylation status in the promoter region of CYP2B1, it has been reported that PB induction modifies the proteins binding to the PBRU region indicating that alterations in chromatin structures including histone acetylation may account for over-expression of CYP2B1^24^,^25^. Agarwal and Shapiro (1996) have suggested that the over-induction of CYP2B1 and 2B2 in adult rats rechallenged with phenobarbital (PB) could be explained by the imprinting of these CYPs^26^. It was suggested that the alterations in growth hormone profiles may result in the over-expression of CYP2B1/2B2 isoenzymes in liver following neonatal exposure of PB^27^. Prenatal exposure of cypermethrin has been shown to alter the circulatory levels of testosterone, growth hormone, follicle stimulating hormone and luteinizing hormone that may also help in explaining the imprinting of these CYPs^11^,^12^,^13^.

As specific MSP primers could not be designed for CYP2B1 due to the complexity of bisulphite converted DNA, bisulphite sequencing incorporating a wider range of promoter sequences led to the identification of critical CpG methylation changes in brain isolated from the challenged offspring. These brain specific changes in CpG methylation were observed in both, basal transcription element (BTE) site, known to control the constitutive expression of CYP2B1 and also in barbiturate responsive BARBIE box, which modulates the response of CYP inducers like PB, located in the promoter sequences of CYP2B1^28^,^29^. It is suggested that the methylation changes observed in BTE and BARBIE box region may transform the binding of transcription factors which in-turn modifies the expression of CYP2B1. As CpG methylation affects the binding of transcription factors to their consensus sequences^30^, the loss of methylation at the CpG, as observed in the present study, may lead to more receptive form of BTE binding site, which may also account for the over-expression of CYP2B1 in brain of challenged offsprings reported earlier^11^,^12^,^13^.

Further evidence that the imprinting of CYPs in brain may enhance the toxicity in the challenged offsprings was provided by our data demonstrating increased apoptosis in these offsprings. A greater magnitude of DNA laddering in the challenged offsprings has suggested increased apoptosis in the challenged offsprings when compared to control offsprings treated postnatally with cypermethrin or prenatally exposed offsprings. The expression profiling of the toxicity pathway genes has indicated induction in the genes responsible for apoptosis in the whole brain isolated from cypermethrin treated mice^31^. Similarly, our western blotting studies also demonstrated significant increase in the protein expression of pro-apoptotic genes such as Bad, Bax, caspase9 and p53 in challenged offsprings suggesting involvement of signalling pathways leading to apoptosis. Although no significant increase was observed in the protein expression of anti-apoptotic gene Bcl-2, our data demonstrating significant increase in the Bax/Bcl-2 ratio in challenged offsprings has indicated increased apoptosis in brain isolated from challenged offsprings as compared to control offsprings treated postnatally with cypermethrin or prenatally exposed offsprings. Previous studies demonstrating greater magnitude of alterations in motor and cognitive functions in challenged offsprings are in further support of the present study indicating that imprinting of CYPs in brain may lead to enhanced toxicity in the rechallenged offsprings^11^,^12^,^32^. Likewise, epigenetic changes leading to greater magnitude of induction in the CYPs may also help in explaining the greater accumulation of cypermethrin and its metabolites in the rechallenged offsprings^14^,^15^.

In conclusion, the present study has demonstrated epigenetic changes involving DNA methylation and histone acetylation in xenobiotic metabolizing CYP1A1 and 2B1 in the brain of rechallenged offsprings. ChIP assay, MSP-UMSP analysis demonstrating alterations in CYP1A1 and 2B1 and specific CpG alterations in the overlapping BARBIE box in CYP2B1 suggests that imprinting of CYP1A1 and CYP2B1 leads to the enhanced responsiveness of cerebral CYP1A1 and CYP2B1 in the rechallenged offsprings during adulthood. Even though the association of CYPs with apoptosis has not been established, the imprinting of CYP1A1 and 2B1 leading to enhanced responsiveness of these CYPs may result in enhanced toxicity in the rechallenged offsprings as evident by increased apoptosis in the brain of rechallenged offsprings. As the exposure to the pyrethroids may occur during pregnancy in agricultural fields, our data assumes significance as imprinting of CYPs may enhance the responsiveness of the individual and predispose them to the toxic effects of centrally acting drugs and chemicals.

## Additional Information

**How to cite this article**: Singh, A. *et al.* Imprinting of cerebral cytochrome P450s in offsprings prenatally exposed to cypermethrin augments toxicity on rechallenge. *Sci. Rep.* **6**, 37426; doi: 10.1038/srep37426 (2016).

**Publisher’s note:** Springer Nature remains neutral with regard to jurisdictional claims in published maps and institutional affiliations.

## Supplementary Material

Supplementary Information
