# Supplementary material for: Imprinting of cerebral cytochrome P450s in offsprings prenatally exposed to cypermethrin augments toxicity on rechallenge
Source: Sci Rep. 2016 Nov 17;6:37426. doi: 10.1038/srep37426 (PMC5112598; doi:10.1038/srep37426)
Supplement: Supplementary Information [file srep37426-s1.pdf]

Supplementary Information

Imprinting of cerebral cytochrome P450s in offsprings prenatally exposed to cypermethrin augments toxicity on rechallenge

Anshuman Singh*(anshuman321_80@hotmail.com), Anita Agrahari*(anuagrahari07@gmail.com), RadhaDutt Singh (radhaduttsingh4@gmail.com), Sanjay Yadav(sanjayitrc@gmail.com), Vikas Srivastava[1] (76.vikas@gmail.com) and Devendra Parmar[1] (parmar_devendra@hotmail.com)

Developmental Toxicology Division, CSIR-Indian Institute of Toxicology Research, Vishvigyan Bhawan, 31 M.G. Marg, Post Box No. 80, Lucknow-226 001, Uttar Pradesh, India

Legend to the supplementary figures

Figure S1a. Densitometric analysis of western blots of total protein isolated from brain of offsprings with anti-Bcl-2/Bax/Bad/caspase 9/p53. All the values represent mean x 10^{-4}±S. E. M. x 10^{-4} of three experiments in each group; *$p<0.05$, **$p<0.01$, ***$p<0.001$. a - compared to control group; b - compared to prenatally exposed offsprings group; c - compared to offsprings exposed postnatally with cypermethin.

Figure S1b. Bar diagram representing % of DNA fragmentation observed in various groups studies from three experiments each; *$p<0.05$, **$p<0.01$, ***$p<0.001$ a - compared to control group; b - compared to prenatally exposed offsprings group; c - compared to offsprings exposed postnatally with cypermethrin.

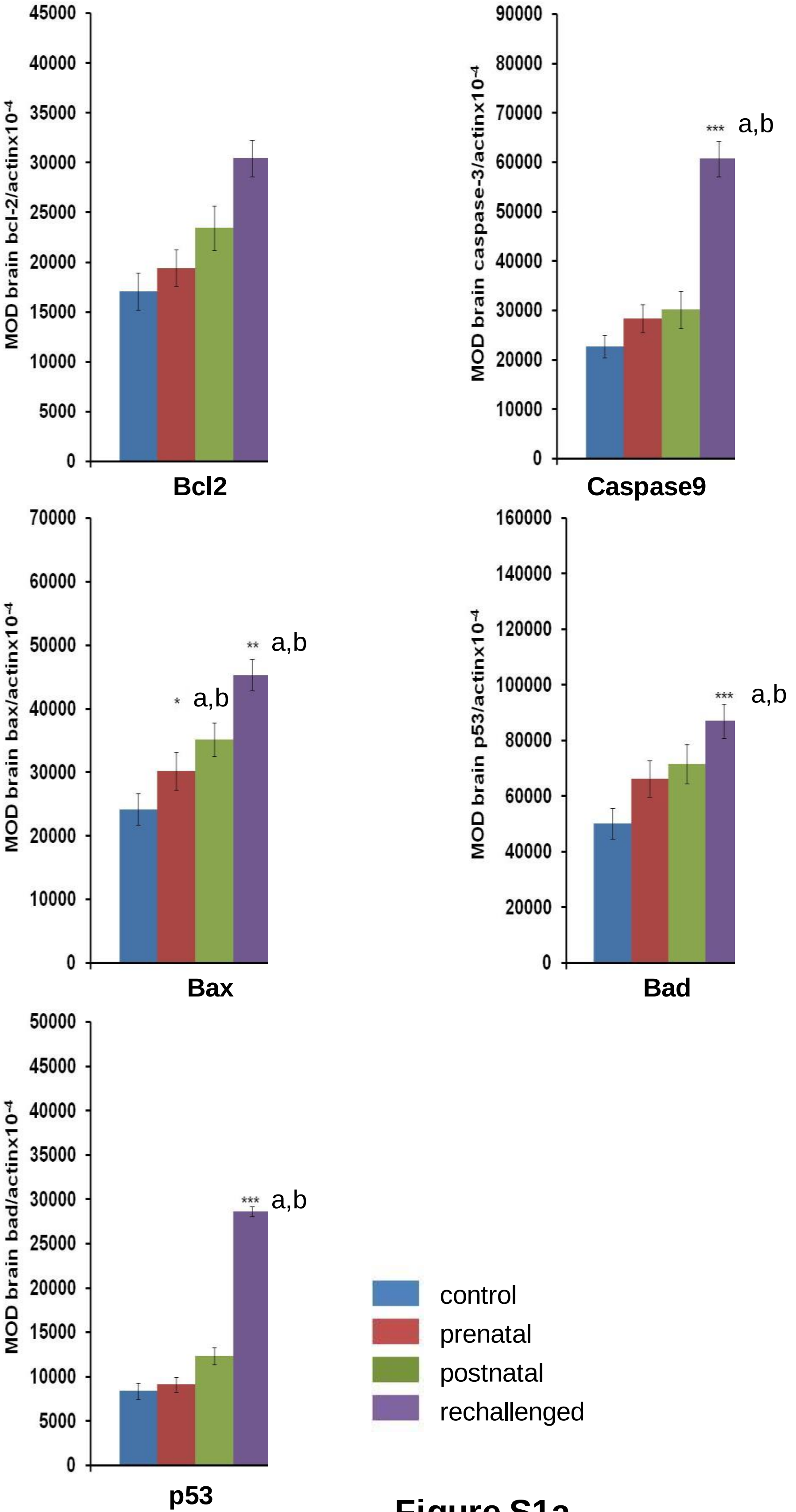
MOD brain bcl-2/actinx10-4
45000
40000
35000
30000
25000
20000
15000
10000
5000
0
Bcl2
MOD brain caspase-3/actinx10-4
90000
80000
70000
60000
50000
40000
30000
20000
10000
0
*** a,b
Caspase9
MOD brain bax/actinx10-4
70000
60000
50000
40000
30000
20000
10000
0
* a,b
** a,b
Bax
MOD brain p53/actinx10-4
160000
140000
120000
100000
80000
60000
40000
20000
0
*** a,b
Bad
MOD brain bad/actinx10-4
50000
45000
40000
35000
30000
25000
20000
15000
10000
5000
0
*** a,b
p53
control
prenatal
postnatal
rechallenged

Figure S1a

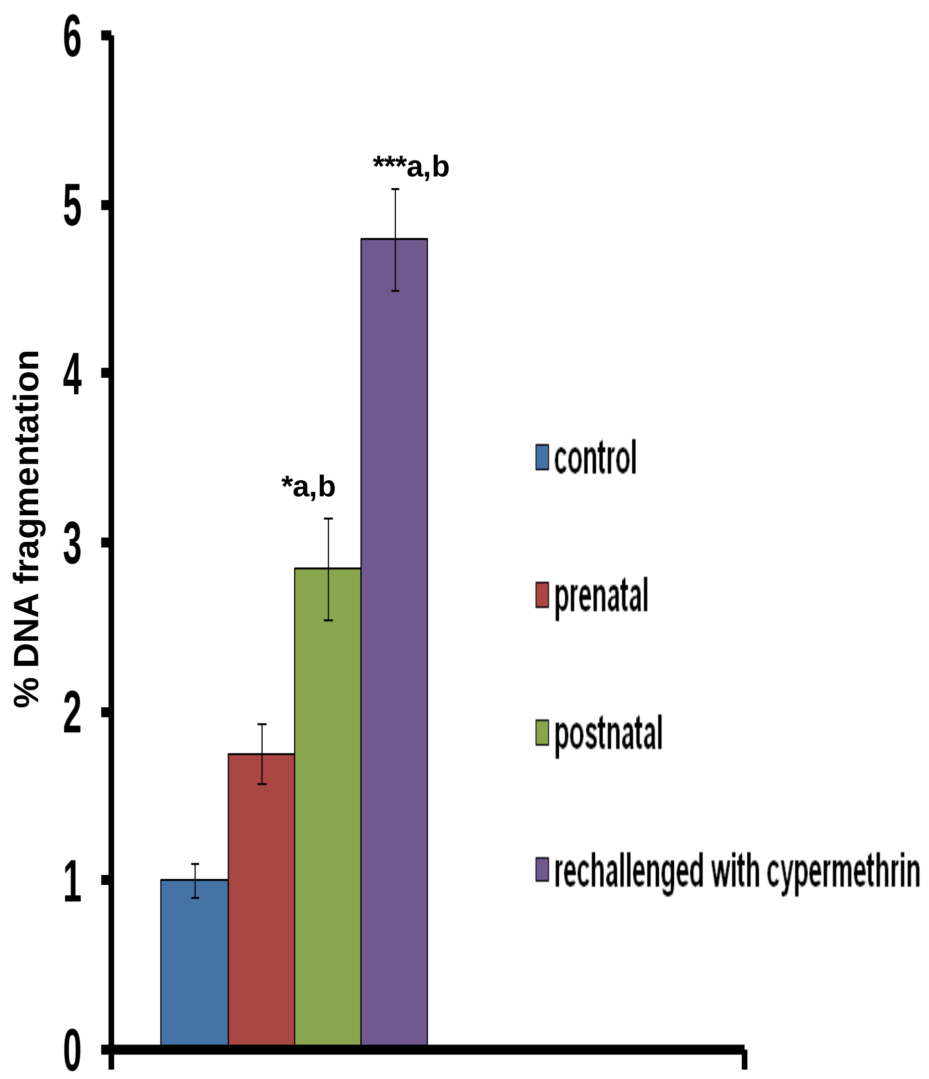
% DNA fragmentation
6
5
4
3
2
1
0
***a,b
*a,b
control
prenatal
postnatal
rechallenged with cypermethrin

Figure S1b

Table S1: Primer's for bisulphite sequencing

Gene Name	Forward Primer	Reverse Primer	Product size	Region
**CYP2B1**	**GTTTTTTTGTTTTGTGTATTTGTTT**	**AACTCCATAATCCTAATATAACCAC**	**202**	**-163 to +38**

All primer sets are in 5' to 3' orientation

Table S2: Primer's for histone and 5 methylcytosine chromatin immunoprecipitation (ChIP) assays

Gene Name (Primer's Name)	Forward Primer	Reverse Primer	Product size	Region
CYP1A1 (R1)	CCTGCAAAACAGCCAGCTA	GTGAGCCTGCTCCATCCTCT	418	-1366 to -948
CYP1A1 (R2)	GAGAGCACCTGCAAAACAGC	GTGAGCCTGCTCCATCCTCT	425	-1373 to -948
CYP1A2 (R3)	TTCAGATGCGTTGGCTCCGA	TGCACATGAATGGGTTGGGC	776	-1323 to -547
CYP1A2 (R4)	TGCCCAACCCATTCATGTGC	ACCCTGTTTCGCTTGCTGTG	664	- 568 to+96
CYP2B1(R5)	TGACCTAGGGGGAAGGTCAGA	GGGAACCCTGAGCTGGACTT	156	-2139 to-1983
CYP2B1 (R6)	GTGACCTAGGGGGAAGGTCAG	AGGAGCAAGGTCCTGGTGTC	113	-2140 to -2027
CYP2B2 (R7)	TCTGGATCGTGGACACAACCT	AGGAGCAAGGTCCTGGTGTC	151	-2177 to -2026
CYP2B2 (R8)	TCTGGATCGTGGACACAACCT	GCACTGTGCCAAGGTCAGGA	115	-2177 to -2062

All primer sets are in 5' to 3' orientation

Table S3: Primer's for methyltion specific PCR (MSP) and unmethylation specific PCR (UMSP)

Gene Name	Forward Primer	Reverse Primer	Product size	Region
**CYP1A1 MSP**	**TATCGTTGGCGTTGTTTAGTC**	**AACTCACTACCGAACGCG**	**165 bp**	**-1240 to -1075**
**CYP1A1 UMSP**	**TTGTATTGTTGGTGTTGTTTAGTT**	**CCCAACTCACTACCAAACACA**	**165bp**	**-1240 to -1075**

All primer sets are in 5' to 3' orientation
